# Gender equality and global health: intersecting political challenges

**DOI:** 10.7189/jogh.10.010701

**Published:** 2020-06

**Authors:** Beniamino Cislaghi, Ann M Weber, Geeta Rao Gupta, Gary L Darmstadt

**Affiliations:** 1 Department of Global Health and Development, London School of Hygiene and Tropical Medicine, London, UK; 2 School of Community Health Sciences, University of Nevada, Reno, Nevada, USA; 3 United Nations Foundation, Washington, D.C., USA; 4 Department of Pediatrics and Center for Population Health Sciences, Stanford University School of Medicine, Stanford, California, USA

## Abstract

**Background:**

Women’s and men’s health outcomes are different. Some differences are biological, related to male and female sex, while others are related to their gender. Sex- and gender-related issues require different solutions, but policy makers lack straightforward heuristic strategies to identify gender-related health inequities.

**Methods:**

Using 169 causes of disability-adjusted life years (DALYs) from the 2017 Global Burden of Disease, we calculated the female-to-male (f:m) and male-to-female (m:f) ratios of global DALYs, rank-ordered the ratios by size and calculated the proportion of all-cause DALYs that each cause explained, separately for males and females 15-49 years old. Gender-related vs sex-related causes were categorised using literature on the drivers for the 15 causes with highest f:m and m:f ratios.

**Results:**

Causes of DALYs with high m:f ratios appear to be gendered and include: road injuries, interpersonal violence, and drowning – totaling 12.4% of men’s (15-49 years) all-cause DALYs. However, causes of DALYs with high f:m ratios are more likely a mix of sex-related and gender-related factors – including headache disorders, depressive disorders, and dietary iron deficiency – totaling 13.4% of women’s (15-49 years) all-cause DALYs. Ratios vary by age, geography and Socio-demographic Index.

**Conclusions:**

Gender-related vs sex-related causes were categorised using available literature on the drivers for selected causes, illustrating that sex-disaggregated data represents a mix of social and biological influences. This analysis offers a model that policy makers can use to uncover potential gender inequalities in health, including intersections with other social factors. From it, new challenges emerge for global health policy makers and practitioners willing to address them. Global health actors will need to achieve a balance between the two agendas of global health and gender equality.

Men live (on average) two to four years less than women, but women experience a higher burden of some morbidities [1]. Important differences in men’s and women’s vulnerability to health-related conditions are not exclusively due to biological (ie, sex-related) causes, but are explained, at least in part, by the influence that gender-related factors have on their choices and actions.

Most of the relevant scholarship shares the premise that gender and sex are different. The word sex refers to the anatomical and physiological traits derived by the composition of sex chromosomes [2,3]. This understanding of sex as biological has been largely accepted and used in the health and medical sciences [4], despite some commentators contesting the biological essence of sex and arguing for its socially constructed nature [5,6]. The word “gender,” on the other hand, is less used – and possibly understood – in the medical scholarship, often seemingly employed as a more academic or elegant word for sex.

Part of the problem with the usage of the word gender is that it has come to represent several, often contradictory, meanings and agendas, such that health policy-makers and practitioners may have different perspectives on what gender is and how it affects the health of both men and women. Two diverging understandings of gender presently exist in the relevant literature. The first sees gender as a psychological identity, one’s internal sense of oneself as a man, woman, transgender or a gender minority; case in point would be, as an example, someone born in a female body who feels they are other than a woman. In the second understanding, gender is a system of expectations and duties that society attaches to that individual because of their sex. In this understanding, for example, women are expected to wear makeup and men to wear ties at important business events not because of their sex (there is no biological reason for them having to dress that way), but because of their gender. In this conceptualisation, gender is a system that assigns expectations, roles, power and ultimately differential access to resources according to whether one is perceived as a man, a woman or other. This “gender system” [7] is embedded within family and social structures in ways that profoundly affect men’s and women’s lives, including their health. In this paper, we specifically use this latter understanding of gender as a system in illustrating its influence on health.

The ways in which the gender system (from now on, just gender) affects people’s health are numerous, with several examples in the literature of, primarily, negative outcomes. Gender affects perinatal care; in certain contexts, for instance, it influences parents’ decision to interrupt a pregnancy when they believe daughters to be a higher economic burden than sons [8]. It affects substance use; heavy drinking, for instance, is often considered a sign of being a ‘real’ man [9]. It affects experience and perpetration of violence: it can contribute to shaping the belief that a woman cannot refuse to have sex with her husband and that he has a right to force her if she does refuse [10]. Not only does gender impact health-related outcomes, it can also influence preventive health behaviours. Globally, men tend to seek less health care than women [11,12] and much less care than women when it comes to accessing, specifically, mental health services [13,14], partly because they are taught that they need to be strong. In contrast, in certain contexts or families, gender can limit women’s decision-making power to go or take her children to the hospital [15], for instance because of limitations placed on women’s mobility outside of the household if not accompanied by their husbands.

While there is some, relatively scattered, empirical evidence of the ways in which gender affects these and other health-related outcomes, many existing studies often focus exclusively on one specific outcome and/or use primary data collected for the study aim, rather than conducting secondary analysis of existing larger data sets (see [16] for a notable exception). To design effective policies at the intersection of gender equality and health, policy-makers need straightforward strategies to uncover gender-related health disparities in national and global data sets containing sex-disaggregated data. In this paper, we offer illustrative insights with both heuristic and procedural value. We provide an indication of the impact that gender can have on people’s health and, at the same time, we offer a methodology that policy-makers and researchers can follow (and, we hope, improve in the future) to uncover similar evidence in their existing data sets. We also offer some final insights into how other social categories [eg, age, geographical residence, and Social-demographic Index (SDI)] intersect with gender in affecting people’s health-related behaviours and vulnerabilities.

## METHODS

### Data source

Sex-disaggregated data for health outcomes were obtained from the Global Burden of Disease Study 2017 (GBD 2017), which includes assessments of the causes and risks of death and disability for 195 countries since 1990. Estimates of disease burden available from the study include number of deaths and death rates, years of life lost due to premature mortality (YLLs), prevalence and prevalence rates for sequelae, years lived with disability (YLDs), and disability-adjusted life years (DALYs), which are the sum of YLLs and YLDs (also referred to as years of healthy life lost). These estimates are modelled based on a large number of input sources (eg, country-level census, vital registrations, disease registries, and local and international surveys) and are updated annually. Detailed descriptions of the methods and approach used for the GBD estimation have been previously described [17]. Data are available disaggregated by sex, age group, country (in some cases by state), and geographic region or demographic country grouping (eg, income levels). Data are open source and can be downloaded from the GBD Results Tool (http://ghdx.healthdata.org/gbd-results-tool).

Disease burden in 2017 was estimated for 359 causes of death and 354 causes of non-fatal health loss, which are categorised into a nested four-level hierarchy. First, causes are split into three large categories (level-one): communicable, maternal, neonatal, and nutritional causes; non-communicable diseases; and injuries. Within each level-one category, causes are broken down by level-two causes, which include 22 broad groupings of conditions (eg, cardiovascular and mental disorders). Level-two conditions are sub-categorised into 169 level-three higher-specificity conditions [eg, stroke (under cardiovascular disorders) and depressive disorders (under mental disorders)]. Some (but not all) level-three conditions are further split to level-four conditions (eg, ischemic stroke and major depressive disorder). Cause lists at each level are mutually exclusive and collectively exhaustive such that causes not individually specified are listed in “other” categories. For this study, we utilised rate estimates of 2017 DALYs per 100 000 population for males and females 15-49 years of age from the 169 level-three causes of disease burden. We analysed DALYs because they represent a comprehensive measure of disease burden, capturing both life and health loss, and we used rates (not numbers) of DALYs for better comparability across countries of varying size. We focused on causes of DALYs for adults 15-49 years of age, excluding causes of early mortality among children, which strongly influence YLLs, and causes of late life morbidity, which strongly influence YLDs for elderly women because of their longer life expectancy. We used level-three causes in order to compare DALYs across a wide-ranging list of specific conditions. Importantly, the methods described here could be applied to other measures of disease burden, age categorisations, categories of conditions, or population sub-groups, although the relative importance of differences in disease burden by sex, and the interpretation of these differences, would change.

### Analysis

To assess the differential burden of disease from specific conditions by sex, we calculated the female-to-male (f:m) and male-to-female (m:f) ratios of global DALYs for all level-three causes. We then rank ordered the ratios by size. We also calculated the proportion of all-cause DALYs that each level-three cause explained, separately for males and females, to estimate each condition’s contribution to the overall burden of disease by sex. For select causes, we repeated the calculations for DALY ratios and sex-specific proportions of all-cause DALYs, disaggregated by 5-year age cohorts and two country groupings: geographic region (using the World Bank categories) and SDI, which combines measures of income per capita, educational attainment for ages 15 years or older, and total fertility rates for women under 25 years. Because this is a descriptive analysis, we did not test whether ratios were significantly different from one.

GBD data have been criticised for multiple reasons: these estimates of disease burden can have large confidence intervals and variations across countries can be affected by the quality of the source data and modelling assumptions in ways that limit comparability [18,19]. Confidence intervals of ratios between data that are imprecise by nature can be subject to further errors. As we mentioned, however, our intent is not to come up with precise estimates. We intend to reflect on these ratios (ie, m:f and f:m DALYs) as illustrations of how sex and gender can interact in affecting health, in a way that can offer an approach which policy-makers can use on more precise data sets.

While in many cases it is difficult to ascertain with absolute certainty the primary cause of a disease, one can still advance an hypothesis informed by the available scientific evidence of which disparities appear to be mostly biologically-determined (ie, related to sex), which ones seem to be primarily socially-driven (ie, gender-related), and which ones are less easily classifiable as attributable to either. Sex and gender are of course interrelated and intertwined, so that health conditions generally are affected by both. Due to the contextual differences across countries (and even subnational regions), the ways in which sex and gender affect people’s health is bound to vary by context. We examine the existing (limited) literature on how sex and gender affected a select number of causes of DALYs that emerged from our GBD analysis to further advance our method and hypotheses for differential impact of sex and gender.

## RESULTS

**Figure 1:**
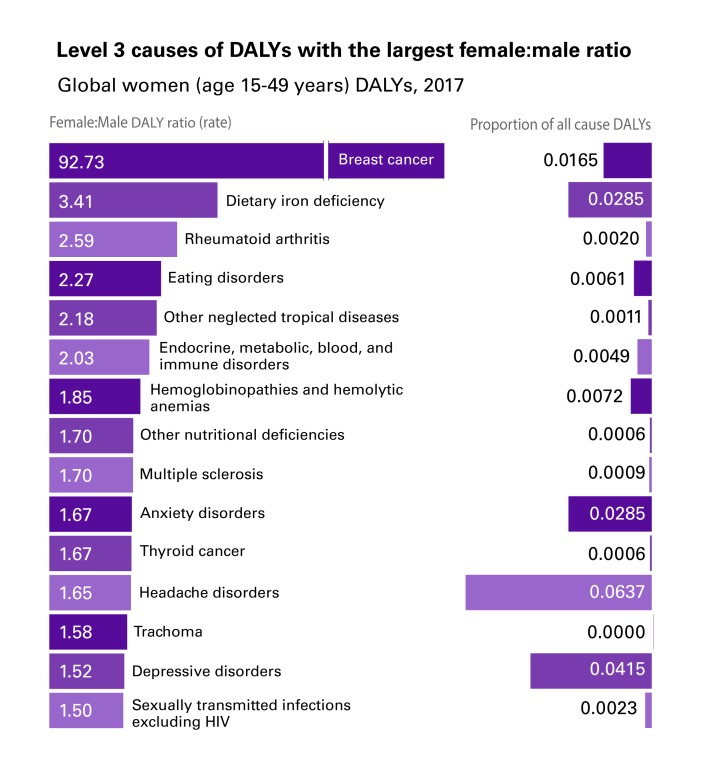
Top-15 disability-adjusted life year (DALY) causes with the largest female:male ratio (15-49 years).

**Figure 2:**
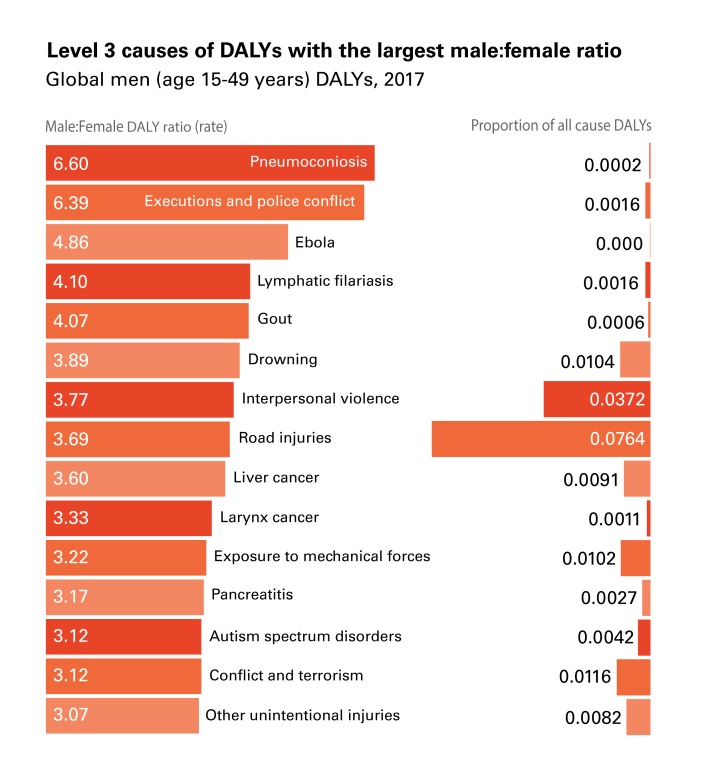
Top-15 disability-adjusted life year (DALY) causes with the largest male:female ratio (15-49 years).

Using the rank-ordered DALY ratios, we identified the 15 largest and non-missing ratios (ratios were missing for sex-specific causes, such as prostate cancer) that disproportionately affected females (**Figure 1**) or males (**Figure 2**) 15-49 years of age globally.

The health conditions in these figures are not (necessarily) men and women’s top-ranking absolute causes of DALYs (ie, the causes with the highest proportion of all causes of DALYs). Rather, they are the top-15 health conditions for which the ratio between the two sexes is the most different from 1. Pneumoconiosis, which ranks first in **Figure 2** (males experiencing 6.6 times the DALYs of females from the condition), explains only 0.02% of men’s all-cause DALYs globally. Other causes in our top-15 are instead important both in absolute and relative terms; for instance, headache disorders explain 6.4% of all-cause DALYs for women and interpersonal violence explains 3.7% of all-cause DALYs for men, while they also have, respectively, a 1.65:1 f:m ratio and a 3.8:1 m:f ratio of global DALYs.

The 92.7:1 f:m DALY ratio from breast cancer, is likely primarily sex-related: vulnerability to breast cancer is made possible by the XX karyotype responsible for the development of breasts [20]. Other disparities, instead, seem to be driven more by gender-related factors. These factors include gender roles dictating different jobs and duties for men and women that explain difference in exposure to relevant determinants. An example is the disproportion with which pneumoconiosis (a respiratory disease) affects men vs women; dust in coal mines increases exponentially the risk of respiratory diseases [21] for miners, in a traditionally male-dominated industry [22,23]. While, biologically, men and women have the same risk of developing pneumoconiosis, men’s risk increases as they access an unsafe environment due to social expectations on the types of jobs that men and women can or should take up. Not only is gender relevant because gender roles and expectations affect exposure to key determinants, gender can also dictate what personality traits ‘real’ men or women should embody. Executions and police conflicts disproportionately affecting men (6.39:1 m:f DALY ratio), for instance, might be related to certain constructions of masculinity that integrate the use of violence and criminality as proofs of one’s manhood [24-28].

Not all causes of DALYs are easily attributable to primarily sex- or gender-related determinants. **Table 1** offers more examples of causes of DALY that seem to be primarily driven by sex or gender, while also including examples of causes of DALY that are either driven by a mix of both or on which the evidence is not yet conclusive. This table is not meant to report definitive results, as both a thorough review of the relevant literature for each of the causes of DALYs goes beyond the scope of this paper, and in some cases the state of evidence is too scarce to come to definite conclusions.

**Table 1:** A hypothesis of whether causes of DALY disproportionately affecting women and men are primarily driven by sex, gender, or both

Cause of DALY	Primary driver of the disparity	Example from the literature
**Women:**		
Breast cancer	Sex	“In the case of cancer it is obvious that only men can get prostate cancer and breast cancer occurs predominantly in women” [20].
Rheumatoid Arthritis	Sex	“RA is a heterogeneous disease with variations in phenotype. Sex-associated factors influence disease severity as well as disease pattern” [29].
Headache disorders	Both or currently difficult to assess	“The nature of women’s pain experiences, their involvement in multiple role responsibilities, and differences in coping strategy utilization likely affect some of the observed sex and gender differences in perception of and responding to pain” [30].
Depressive disorders	Both or currently difficult to assess	“Generally, only limited evidence exists for gendered risk factors to be specific for depression [but] findings from cross-national studies suggest a temporal trend of a converging gender gap in depressive disorders in countries with less traditional gender roles” [31].
Trachoma	Gender	“Women and girls are the primary caregivers in most societies in developing countries. Proximity to children exposes women to repeated infection more than men and is likely a primary reason for the greater effect of active disease” [32].
Eating Disorders	Gender	“Eating disorders are more prevalent in the female because more of them are dieting to lose or control weight. Their greater dysphoria, poorer self-image and body concept, and role confusion during adolescence also appear to contribute to the risk of developing an eating disorder because these factors intensify their pursuit of thinness” [33].
**Men:**		
Mesothelioma	Sex	“The differential expression of steroid hormone receptors between sexes … may be suspected to play a role in the cancer biology of peritoneal mesothelioma” [34].
Bladder Cancer	Sex	“Difference in bladder cancer incidence is independent of differences in exposure risk … the activity of the sex steroid hormone pathway may play a role in bladder cancer development, with demonstration that both androgens and estrogens have biologic effects in bladder cancer in vitro and in vivo” [35].
Gout	Both/Difficult to assess	“Gouty arthritis occurs more frequently in men at younger ages than in women and is regarded as a result of interplays between genetic, metabolic, and environmental factors.” [36].
Autism spectrum disorders	Both/Difficult to assess	“Accurate and early diagnosis of autism in both sexes is essential, not only for understanding sex differences in ASD, but also for providing appropriate resources and services. Accurate diagnosis and identification of autism-like features is also necessary for lifelong support of women whose impairments may be traditionally under recognized. These advancements will require further research and scientific study” [37]
Interpersonal Violence	Gender (sex might predispose men)	“Physical dominance and violence are easily accessible resources for structuring, negotiating and sustaining masculinities, particularly among men who because of their social positioning lack less dangerous means” [38]
Alcohol use disorders	Gender (Sex disadvantages women)	“Presented review of the contemporary evidence supports the presence of both gender- and sex-related differences in alcohol use… Gender-specific differences in alcohol consumption … are driven by … perceived differences in traditional gender roles in different countries and among different generations… Differences in alcohol consumption, elimination, and distribution volume, result in higher blood alcohol concentrations in women, which also tend to persist longer compared to men” [39].

DALY – disability-adjusted life year

The illustrative findings presented in the table above offer an opportunity for further reflection on the differences between sex and gender. Consider, for instance, the evidence suggesting that Trachoma and Alcohol use disorders are largely driven by gender-related factors. Trachoma – an infection caused by the *Chlamydia trachomatis* bacterium – is the leading cause of infectious blindness [40]; the most recent data (2003) estimate 84 million people worldwide are actively living with this condition. The disease is preventable with simple hygiene practices (eg, daily face washing). A single infection episode is usually cleared by a person’s immune system; repeated infections, however, lead to scarring of the cornea and eventually to visual impairment or blindness. From a biological standpoint, both men and women are equally vulnerable to the infection, but women get infected two to three times more frequently [32]. Reasons for this disparity are thus to be sought in gender roles that assign child-caring duties to women: primary-school children are a major vector for the bacterium, with infections among children being very common.

Alcohol use disorders, in contrast, involve interactions of gender-related factors with people’s sex-related biological characteristics. Evidence suggests that women are at greater risk than man of alcohol-induced cardiomyopathy [41], peripheral neuropathy [42], volumetric brain loss [40], and severe alcoholic liver problems [43]. Despite women’s greater vulnerability, however, alcohol use disorders disproportionately affect men. Trying to understand the motivation for this disparity, in a comprehensive review, Erol and Karpyak [39] found that: “Reports from multiple countries located on different continents indicate that men consume more alcohol than women, drink more frequently, and are more likely to be hazardous drinkers” (p.3). A key reason for men’s higher consumption is traditional gender roles that reward drinking among men and sanction drinking among women. While men who drink are more likely to be respected [44] or considered ‘macho’ by their friends [9]. women who drink are at risk of being ostracised [45]. In recent decades, however, these differences have begun to attenuate, especially among youth living in high income countries [39].

### Intersectional case studies

While the focus of this paper is on the necessity of integrating gender in public health analysis and policy-making, it is also important to acknowledge that the explanatory potential of gender as a single analytical category has serious limitations. These limitations have been stressed by proponents of the “intersectionality theory,” who argued that other social categories, such as ethnicity, class, ability, age, sexual orientation, and geography intersect with gender in creating social vulnerabilities and hierarchies of access to services and resources [46,47]. We provide further illustration of the ways in which gender intersects with other social categories, examining two case studies among the causes of DALYs with the highest f:m and m:f ratios in **Figures 1** and **Figure 2**, respectively: 1) Eating disorders, and 2) Road Injuries.

### Eating disorders

**Figure 3:**
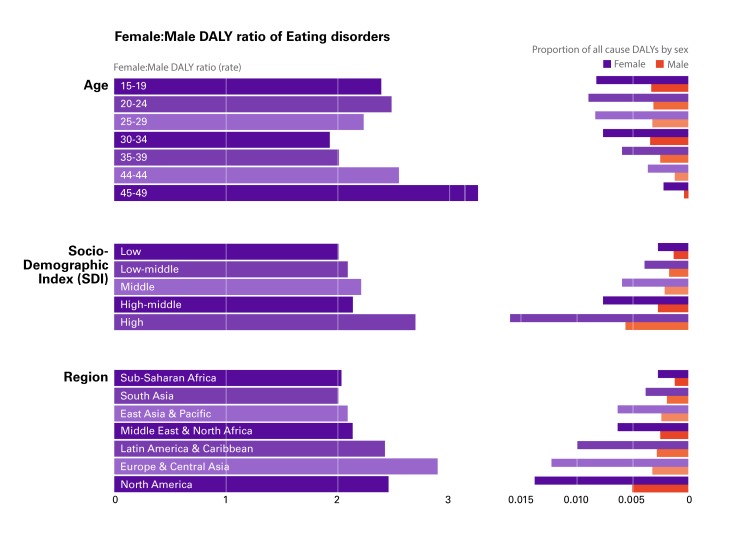
Female:Male disability-adjusted life year (DALY) ratio for eating disorders.

Eating disorders (Anorexia and Bulimia Nervosa) account for 3.2 million (2.1-4.7 million) DALYs, 0.40% of total global DALYs for both males and female 15-49 years in the 2017 GBD. The rate of eating disorder DALYs for both sexes increased in the last two decades, from 71.8 per 100 000 (45.9-104.6) in 1997 to 82.5 (52.7-119.9) in 2017. The 2.3:1 f:m disparity in eating disorders has been explained by highly-gendered feelings of body satisfaction: the more people are dissatisfied with their body’s size or shape, the more likely they are to experience an eating disorder [48,49]. The greater preponderance of these disorders in women is well-established in the literature, with some commentators suggesting that “Few psychiatric disorders are as sexually dimorphic as the eating disorders” [50]. GBD data provides indication of the sexually dimorphic nature of eating disorders, but it also illustrates differences in the f:m disparity across ages, geographies, and Socio-Development Index (SDI) (**Figure 3**).

During adolescence (ages 15-19 years) and young adulthood (20-24 years) eating disorders have a wide f:m disparity (ratios 2.4:1 and 2.5:1, respectively) and explain nearly 1% of all-cause DALYs for those two age groups (0.82% and 0.89%, respectively). The f:m disparity is even wider later in adulthood (40-44 years: 2.5:1; and 45-49 years: 3.3:1) but with much smaller contributions to all-cause DALYs for women in these age groups (0.36% and 0.22%, respectively). In geographic terms, the disparity is widest in the three regions of Latin America & Caribbean (2.4:1), North America (2.5:1) and Europe & Central Asia (2.9:1); three regions where the contribution to all-cause DALYs for women is also the highest (0.99%, 1.4%, and 1.2%, respectively). SDI differences, finally, are the most pronounced. Here, the f:m disparity is the widest in High SDI countries (2.7:1), where eating disorders contribute to 1.6% of all-cause DALYs for women.

### Road injuries

**Figure 4:**
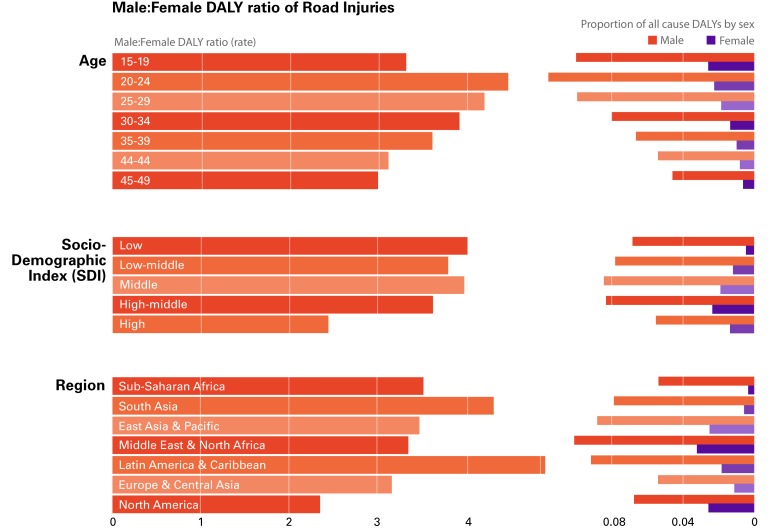
Male:Female disability-adjusted life year (DALY) ratio for road injuries.

Road injuries, the leading cause of preventable death [51], account for 42.1 million (40.1-44.0 million) DALYs, 5.26% of total global DALYs for both males and female 15-49 years in the 2017 GBD. The rate of DALYs from road injuries for both sexes decreased considerably in the last two decades, from 1490.3 per 100 000 (1434.7-1549.9) in 1997, to 1076.0 per 100 000 (1026.4-1124.2) in 2017. The 3.7:1 disparity in road injuries between men and women has been repeatedly observed cross-culturally [52-55], but less evidence is available on the role that gender, specifically, plays in road injuries. Commentators have suggested the disparity is due to: 1) differential access to driving, and 2) gender-related hazardous behaviour in men. In some countries, women were not legally allowed to drive until recently and, even now that they are, gender norms (gender-related expectations of appropriate behaviour) make it inappropriate for them to do so [56]. At the same time, in certain contexts, gender norms dictate that men should take risks in disregard of possible injury, potentially leading to hazardous driving behaviour [52]. The GBD data further provide evidence of how age, geographical location and SDI affect the m:f disparity (**Figure 4**).

Young adults (20-24 years) have the widest m:f disparity (4.5:1), explaining 11.6% of men’s all-cause DALYs. The latter is unsurprising, given that road traffic injury is the leading cause of death for those aged 5–29 years [51]. The magnitude of the m:f disparity in this age group is probably related to the fact that, during adolescence and young adulthood, norms tend to dictate that men, in particular, prove their masculinity through hazardous and risky behaviours [56]. Conversely, the lower disparity in high SDI countries (2.4:1) and lower proportion of all-cause DALYs (5.5%) compared to lower SDI countries might be due to better infrastructure, driving law enforcement systems and safety devices such as seatbelts and airbags in cars [51]. Latin American & Caribbean and South Asia are the two geographic regions with the highest disparity (4.9:1 and 4.3:1), despite the fact that the highest proportional contribution of road accidents to all-cause DALYs among men 15-49 years is found in the Middle East and North Africa region (10.1%).

## DISCUSSION

In this paper, we used third-level causes of DALYs from the 2017 GBD database to identify the top-15 causes of DALYs that disproportionately affect women and men. We suggested that reasons for these disparities vary, and that, while some are sex-driven, others are driven by the gender system. We have also used two case studies, eating disorders and road injuries, to provide evidence of how gender intersects with other social categories (age, geography, and SDI) in affecting disparities in causes of DALYs.

While our analysis offers a series of illustrations to advance the discussion in the medical sciences on the differences between sex and gender, it should be recognised that some health-related outcomes are influenced by both sex and gender, albeit with the two playing a different role by context. Furthermore, our analysis of the sex- and gender-related factors for each cause of DALYs does not intend to be definitive, but rather to open up the discussion on the differences between gender and sex, their medical implications, and possible areas of expansion of existing public health efforts. In the case of alcohol disorders and intimate partner violence, for instance, purposeful interventions might integrate strategies to target masculinity norms that make drinking and fighting symbols of being a ‘real’ man. Similarly, for eating disorders, policy makers might want to include in their work strategies to relieve women (and men) from the pressure of achieving a body shape modelled on unrealistic social expectations. While great medical and scientific advancements are making available to policy makers more and more effective technological solutions, our analysis suggests that some health-related conditions cannot be solved by those solutions alone; they require strategies that also target social drivers of ill-health. We hope that others in the medical sciences will join this research trajectory and conduct more empirical studies or relevant reviews.

As policy makers identify possible pockets of health-related gender disadvantages that need addressing, their decisions will need to exit the value-neutral space of technical policies to enter the heavily political space of social justice. Take, for instance, the case of trachoma. Here, the f:m disparity is partly related to the fact that women are traditionally assigned roles of caregivers of children. Policy-makers will need to decide whether they want to use this evidence, for instance, to raise awareness among women of the risks related to trachoma, or to rebalance distribution of labour among men and women (possibly landing on a mix of initiatives that include both strategies).

From this analysis there are four key messages that we offer to global health policy-makers. The first is: *It’s not just about biology*. Sex and the gender system both operate to influence people’s health, access to treatment and preventive behaviours. In this paper, we provided a simple analytical method to identify health outcomes potentially affected by gender-related factors that deserve to be studied further for effective improvement of those outcomes. The second key message is: *It’s not just about women*. The word gender has traditionally been associated with women and girls, so that men’s gender-related health needs have been relatively understudied. Gender affects both men and women, in protective as well as risky and harmful ways. We believe that effective policies could help people embody their genders in ways that allow them to take better care of themselves and others. The third key message is: *It’s not just about gender*. As we suggested in two case studies, gender intersects with other social categories that policy makers need to take into account in their work. A white upper-class middle-age woman might have (and probably has) better access to health care than a black working-class elderly man. The final key message is: *It’s not just about prevention and treatment*. As policy makers begin to work to address pockets of health disparities, which is foundational to the achievement of the Sustainable Development Goals, their actions enter into the moral realm of political and ethical decisions in ways that place social justice at the centre of their work.

## CONCLUSIONS

In this paper we used GBD sex-disaggregated data to provide evidence of the need to further understand how social and biological drivers interact in affecting women’s and men’s health. Our goal was also to provide an approach that policy makers can use to begin to untangle the differential roles that sex and gender play in affecting health to enable them to develop more effective policies in their particular context to address the health of populations. We did not aim to provide solutions, rather to reframe the questions that shape data interrogation and implementation strategies. As scientific progress increases our ability to address the more technical-related issues, we are left with the need to address, specifically, the social barriers to greater health and well-being for all. As policy-makers do so, they will need to deal with value-based political decisions that will require transparent engagement with the population reached by their policies and interventions. Future research methods might help to better quantify the contribution of gender, sex and other intersecting factors to any given health outcome. Until then, greater cross-disciplinary mixed-method research can point us towards future questions and learnings.
